# Typing of *Borrelia* Relapsing Fever Group Strains

**DOI:** 10.3201/eid1009.040236

**Published:** 2004-09

**Authors:** Jonas Bunikis, Jean Tsao, Ulf Garpmo, Johan Berglund, Durland Fish, Alan G. Barbour

**Affiliations:** * University of California—Irvine, Irvine, California, USA;; † Yale University School of Medicine, New Haven, Connecticut, USA;; ‡ Michigan State University, East Lansing, Michigan, USA;; § Kalmar County Hospital, Kalmar, Sweden

**Keywords:** Borrelia infections, Lyme disease, relapsing fever, soft ticks, hard ticks, rRNA operon, intergenic region, p66 outer membrane protein gene, dispatch

## Abstract

Partial sequencing of the 16S-23S rDNA intergenic spacer showed two to four genotypes each for *Borrelia hermsii* and *B. turicatae*, both relapsing fever agents transmitted by argasid ticks, and for *B. miyamotoi* and *B. lonestari*, transmitted by ixodid ticks. Field surveys of *Ixodes* ticks in Connecticut and Sweden showed limited local diversity for *B. miyamotoi*.

The two major clades of species in the genus *Borrelia* are the Lyme borreliosis group and the relapsing fever group (*1*). The Lyme borreliosis group includes *Borrelia burgdorferi*, *B. afzelii*, *B. garinii*, and several other species not associated with human disease. The relapsing fever group includes several species, such as *B. hermsii* in the Nearctic ecologic region and *B. persica* in the Palearctic, that cause endemic relapsing fever in humans (*2*). The known relapsing fever agents are transmitted by soft (argasid) ticks, usually an *Ornithodoros* species. In 1995, *B. miyamotoi* was first isolated from *Ixodes persulcatus* hard (ixodid) ticks in Japan (*3*). Genomic DNA of the newly identified spirochete cross-hybridized to a greater extent with DNA of relapsing fever species than with DNA of Lyme borreliosis species. In 1996, *B. lonestari* was discovered in *Amblyomma americanum*, an ixodid tick of the southern and eastern United States (*4**,**5*). Although *B. lonestari* is associated with a Lyme borreliosis–like disorder in the southern United States (*6*), sequence analysis showed that *B. lonestari*, like *B. miyamotoi*, was in a clade with the relapsing fever group rather than the Lyme borreliosis group (*4**,**5*). More recently, spirochetes closely related to *B. miyamotoi*, and provisionally designated here as *B. miyamotoi* sensu lato (s.l.), were discovered in *I. scapularis* ticks in the United States (*7*) and *I. ricinus* ticks in Europe (*8*).

## The Study

The public health importance of the newly discovered species remains to be determined. However, finding *B. miyamotoi* s.l. in *I. scapularis*, *I. ricinus*, and *I. persulcatus*, the predominant vectors of Lyme borreliosis in North America, Europe, and Asia, respectively, complicates interpreting epidemiologic studies of Lyme borreliosis and other ixodid-borne disorders. A method to identify and distinguish strains within species is needed to carry out studies of the population biology and of the possible etiologic roles of these organisms. Since most of these microorganisms are to date uncultivable or poorly cultivable, a method using DNA amplification by polymerase chain reaction (PCR) is preferable. On the basis of the findings of Liveris et al. (*9*), we further developed sequence analysis of the 16S-23S rRNA intergenic spacer (IGS) for strain typing and showed its advantages over other loci for the Lyme borreliosis agents *B. burgdorferi* and *B. afzelii* (*10*). For this study, we applied this approach to typing the new *Borrelia* spp. and included two relapsing fever agents, *B. hermsii* (endemic in the western and northwestern United States) and *B. turicatae* (endemic in the southwestern and south-central United States) (*2*).

Nine isolates of *B. hermsii* in our culture collection originated in New Mexico, Colorado, California, and Washington State and were either from *Ornithodoros hermsi* ticks, patients with relapsing fever, or, in one case, a bird (*11*). Two *B. turicatae* isolates were from *O. turicata* ticks from Texas and Kansas. *B. miyamotoi* strains HT24, HT31, and HK004 from *I. persulcatus* ticks and strains NB103-1 and FR64b from *Apodemus* spp. mice were from Hokkaido, Japan (*3**,**12*). Cultivable strains of these species were grown in Barbour-Stoenner-Kelly II medium. Uncultivated species were initially identified in total DNA extracts of ticks by using *Borrelia* genus–specific PCR, targeting *flaB* gene (*5*). Approximately 2% of *A. americanum* nymphs and adult females in collections from different areas of New Jersey, Illinois, and Missouri contained *B. lonestari*. *B. miyamotoi* s.l. spirochetes were identified in *I. scapularis* nymphs collected at a 7.2-ha field site in southern Connecticut and in *I. ricinus* nymphs collected at a 1.5-ha site in Blekinge County in Sweden (*10*). A Connecticut strain of *B. miyamotoi* s.l. strain was maintained in *Mus musculus* (*7*).

Part of the intergenic spacer was amplified by PCR with primers for the 3´ end of the 16S rRNA gene and the *ileT* tRNA gene (*10*). As a comparison to the intergenic spacer locus and to assess linkage disequilibrium, we also partially sequenced the chromosomal gene for the P66 outer membrane protein (*10**,**13*) after amplification by PCR as described in the Table footnotes. The PCR products were either directly sequenced or first cloned into pCR2.1-TOPO vector (Invitrogen, Carlsbad, CA) before sequencing on a Beckman CEQ 8000 (Beckman Coulter, Fullerton, CA) automated sequencer. The sequences were aligned automatically by using Clustal X software (http://www-igbmc.u-strasbg.fr/BioInfo/ClustalX) and then manually with MacCLADE version 4.05 (http://macclade.org/macclade.html) (*10*). The maximum lengths of the alignments (http://spiro.mmg.uci.edu/data) were set by the shortest available sequence. Accession numbers for the deposited sequences are given in the legend for the Figure and in a footnote for the Table.

**Table:** Descriptive statistics for IGS^a^ and *p66* loci of three *Borrelia* species

Species	Locus	No. samples	No. variants	Aligned characters			
				Base pairs	No. gapped	Polymorphisms (%)	π^b^
*Borrelia miyamotoi* s.l.	IGS	33	4	474	15^c^	40 (8.4)	0.058
	*p66* ^d^	19	3	617	9	38 (6.2)	0.042
*B. lonestari*	IGS	20	3	412	1	14 (3.4)	0.023
	*p66* ^e^	7	3	346	0	5 (1.4)	0.010
*B. hermsii*	IGS	9	4	665	2	20 (3.0)	0.015
	*p66* ^e^	5	3	516	3	8 (1.6)	0.010

^a^IGS, 16S-23S rRNA gene intergenic spacer region. ^b^π, mean nucleotide diversity at each aligned position. ^c^Excludes an 81-bp indel. ^d^Partial *p66* genes were amplified by nested PCR with outer forward and reverse primers of 5´GATTTTTCTATATTTGGACACAT and 5´AATTTAATCAGATTGTTTAGCTCTA and inner primers of 5´GACACATATCTAAAAAAGCAAACAC and 5´CTAATCCGGTTTTTACGTATATGC and following conditions: 40 cycles of 94°C for 60 s, 55°C for 120 s, and 74°C for 120 s. GenBank accession numbers for *p66* genotypes are the following: *B. miyamotoi* s.l. type 1 (AY363722), type 2 (AY363723), type 3 (AY363724); *B. lonestari* type 1 (AY363689), type 2 (AY363690), type 3 (AY363691); *B. hermsii* type 1 (AF016408), type 2 (AF228028), and type 4 (AF116905). ^e^Analysis of *B. lonestari* and *B. hermsii* *p66* fragments was limited to 346 of 605 bp and 516 of 608 bp, respectively, which corresponded to the shortest sequences available for all types of the individual species.

The PCR products for the intergenic spacer locus varied in length between species and ranged from 388 bp for *B. miyamotoi* s.l. from Sweden to 685 bp for *B. turicatae*. The PCR product for the *p66* gene was 605–614 bp between species. The Table summarizes the statistics for the aligned intergenic spacer and *p66* sequences of the *B. miyamotoi* s.l., *B. lonestari*, and *B. hermsii*. The mean nucleotide diversity normalized for each aligned position was 38%-130% higher for the intergenic spacer locus than for the *p66* locus. At the same time, intragenic recombination was not detected at the intergenic spacer locus with Sawyer's test (www.math.wustl.edu/~sawyer/mbprogs), which assesses the likelihood that polymorphisms in a sequence arose through recombination rather than mutation (data not shown). This result was consistent with the undetectable recombination at the intergenic spacer loci of *B. burgdorferi* (*10*).

The genetic diversity at the intergenic spacer and *p66* loci for the relapsing fever group species in a given geographic area was more limited than was the case for Lyme borreliosis species (*10*). This limitation was most apparent with the *B. miyamotoi* s.l. sequences of 22 samples from Connecticut and 6 samples from Sweden. As shown by the phylogram (Figure), only one intergenic spacer genotype each was found for *B. miyamotoi* s.l. from the Connecticut site and from Sweden. In contrast, collections at the same sites and times, and from the same tick vectors, provided 8 intergenic spacer genotypes among 62 *B. burgdorferi* samples in *I. scapularis* and 9 intergenic spacer genotypes among 73 *B. afzelii* samples in *I. ricinus* (*10*). Accepting a type I error level of 0.05, we would expect to have detected a second genotype of *B. miyamotoi* s.l. in a sample size of 22 if its proportion was >14%. The findings at the *p66* locus for 10 Connecticut samples and for 4 samples from Sweden were similar: only one *p66* genotype was detected at each location.

**Figure:**
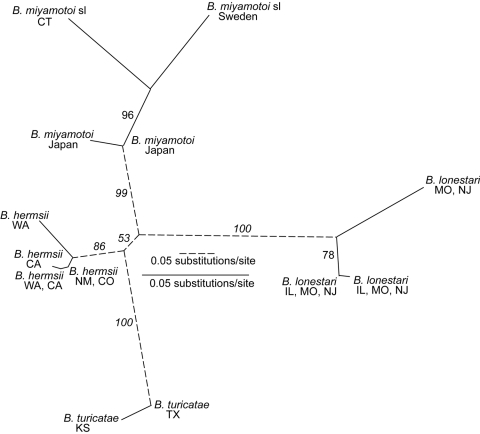
Unrooted maximum-likelihood phylogram for 16S-23S ribosomal RNA gene intergenic spacer sequences of *Borrelia miyamotoi* s.l., *B. lonestari*, *B. hermsii*, and *B. turicatae*. Maximum likelihood settings for version 4.10b of PAUP* (http://paup.csit.fsu.edu) for equally weighted characters corresponded to Hasegawa-Kishino-Yano model with transition/transversion ratio, nucleotide frequencies, proportion of invariable sites, and gamma distribution shape parameter estimated by maximum likelihood. Support for clades was evaluated by 100 bootstrap replications using full-heuristic search, and values >50% are indicated along branches. Solid and dashed scale bars indicate the number of substitutions per site for the corresponding branches. The geographic origins of the isolates are indicated. The GenBank accession numbers for IGS 16-S rRNA gene intergenic spacer genotype sequences are as follows: *B. miyamotoi* s.l. type 1 (AY363703), type 2 (AY363704), type 3 (AY363705), and type 4 (AY363706); *B. lonestari* type 1 (AY363707), type 2 (AY363708), and type 3 (AY363709); *B. hermsii* type 1 (AY515265), type 2 (AY515266), type 3 (AY515267), and type 4 (AY515269); *B. turicatae* type 1 (AY526494) and type 2 (AY526495). WA, Washington State; CA, California; NM, New Mexico; CO, Colorado; KS, Kansas; TX, Texas; IL, Illinois; MO, Missouri; NJ, New Jersey.

The samples of the other relapsing fever group species were not prospectively acquired for population studies, and thus, the findings provide only a tentative view of population structure. Nevertheless, the results are consistent with an interpretation that the local strain diversity of the relapsing fever group species is more limited than that of Lyme borreliosis agents. The intergenic spacer sequences of five *B. miyamotoi* isolates from ticks or mice from Japan were identical, except for a single nucleotide in one isolate (Figure); the *p66* sequences were identical for each of the five isolates. Four intergenic spacer genotypes were detected from the nine isolates of *B. hermsii* from different regions of the western United States; the three intergenic spacer genotypes that were examined each had a different *p66* allele. Two of the linked intergenic spacer and *p66* genotypes were unique to species from the Rocky Mountain region. The two strains of *B. turicatae* from Texas and Kansas differed in intergenic spacer genotype. *A. americanum* ticks collected in three states yielded three intergenic spacer genotypes from 20 samples positive for *B. lonestari* (Table and Figure). The three intergenic spacer genotypes were each linked to three unique *p66* alleles. Two of the linked genotypes were found at all three locations; one was found in Missouri and New Jersey but not in Illinois.

## Conclusions

Samples of the *B. miyamotoi* s.l. showed greater genetic diversity at the intergenic spacer locus than did samples from other genomic groups (Figure). However, even for species with fewer polymorphisms (Table), the intergenic spacer sequences, with or without the *p66* sequences, confirmed the monophyly of strains within each species. This pattern of relationship and the lack of evidence of gene conversion from horizontal gene transfer at this locus, demonstrates that, as for the Lyme borreliosis spirochetes (*10*), the intergenic spacer region is both sensitive and sufficient for genotyping the relapsing fever group of *Borrelia* species. Sequencing this locus provides a means for further epidemiologic and ecologic studies of the newly discovered *Borrelia* species of hard ticks, as well as of the relapsing fever agents that are reemerging as human pathogens (*1*). Certain intergenic spacer genotypes of *B. burgdorferi* are associated with certain virulence phenotypes in humans (*14*). Strain typing by PCR and sequence analysis should also be useful for identifying and characterizing the vertebrate reservoirs of *B. lonestari* and *B. miyamotoi* s.l.

The linkage disequilibrium between the intergenic spacer and *p66* loci indicate that the relapsing fever group species, like Lyme borreliosis spirochetes (*10**,**15*), are highly clonal bacteria. Why these two groups of tick-borne spirochetes appear to have different population structures remains to be determined. This variation may be the consequence of differences in pathogenesis between the organisms.
